# List of Accidents Admitted into the Pennsylvania Hospital, from Dec. 27, 1837, to Jan. 10, 1838

**Published:** 1838-01-17

**Authors:** 


### HOSPITAL REPORTS.

*List of accidents admitted into the Pennsylvania Hospital, from Dec.* 27, 1837, *to Jan.* 10, 1838.

One fracture of the tibia and fibula; two lacerated wounds of the head, since discharged, cured; one gunshot, and one lacerated wound of the hand; a contusion of the abdomen; and a rupture of a varicose vein, in a female, since discharged, cured.

The fracture of the forearm, reported last week, has been discharged, cured in 24 days. In the case of the "lacerated wound in the sole of the foot, caused by a locomotive," mortification ensued, and the man died, 16 days after admission.

